# Conditional cash transfer schemes in Nigeria: potential gains for maternal and child health service uptake in a national pilot programme

**DOI:** 10.1186/s12884-014-0408-9

**Published:** 2014-12-12

**Authors:** Ugo Okoli, Laura Morris, Adetokunbo Oshin, Muhammad A Pate, Chidimma Aigbe, Ado Muhammad

**Affiliations:** SURE-P MCH Project Implementation Unit, National Primary Health Care Development Agency, Abuja, Nigeria; Duke Global Health Institute, Duke University, Durham, USA; National Primary Health Care Development Agency, Abuja, Nigeria

**Keywords:** Antenatal care, Conditional cash transfer, Demand creation, Health services, Maternal health, Maternal newborn and child health, Nigeria, Rural areas, Skilled birth attendance, Social protection

## Abstract

**Background:**

This paper describes use of a Conditional Cash Transfer (CCT) programme to encourage use of critical MNCH services among rural women in Nigeria.

**Methods:**

The CCT programme was first implemented as a pilot in 37 primary health care facilities (PHCs), in nine Nigerian states. The programme entitles women using these facilities up to N5,000 (approximately US$30) if they attend antenatal care (ANC), skilled delivery, and postnatal care. There are 88 other PHCs from these nine states included in this study, which implemented a standard package of supply upgrades without the CCT. Data on monthly service uptake throughout the continuum of care was collected at 124 facilities during quarterly monitoring visits. An interrupted time series using segmented linear regression was applied to estimate separately the effects of the CCT programme and supply package on service uptake.

**Results:**

From April 2013-March 2014, 20,133 women enrolled in the CCT. Sixty-four percent of beneficiaries returned at least once after registration, and 80% of women delivering with skilled attendance returned after delivery. The CCT intervention is associated with a statistically significant increase in the monthly number of women attending four or more ANC visits (increase of 15.12 visits per 100,000 catchment population, p < 0.01; 95% confidence interval 7.38 to 22.85), despite a negative level effect immediately after the intervention began (-45.53/100,000 catchment population; p < 0.05; 95% CI −82.71 to −8.36). A statistically significant increase was also observed in the monthly number of women receiving two or more Tetanus toxoid doses during pregnancy (21.65/100,000 catchment population; p < 0.01; 95% CI 9.23 to 34.08). Changes for other outcomes with the CCT intervention (number of women attending first ANC visit; number of deliveries with skilled attendance; number of neonates receiving OPV at birth) were not found to be statistically significant.

**Conclusions:**

The results show that the CCT intervention is capable of significant effects on service uptake, although results for several outcomes of interest were inconclusive. Key lessons learnt from the pilot phase of implementation include a need to track beneficiary retention throughout the continuum of care as closely as possible, and avert loss to follow-up.

## Background

### Introduction

Nigeria, the most populous country in Africa, has struggled over past decades to improve health outcomes for approximately 39 million women of childbearing age and 30 million children under the age of five^a^ [1,2]. An estimated 40,000 maternal deaths occur annually in Nigeria, comprising 14% of the global burden of maternal mortality [3]. The maternal mortality ratio is estimated at 576 per 100,000 live births, far from the Millennium Development Goals target of 275/100,000 by 2015 and with no statistically significant change since 2008 [1].

Given Nigeria’s contribution to the burden of mortality and the urgent need to reduce these figures, new and innovative approaches to improve maternal, neonatal and child health (MNCH) deserve attention. This paper provides an early description and discussion of one such intervention by the Federal Government of Nigeria’s Subsidy Reinvestment and Empowerment Programme (SURE-P): the pilot phase of a Conditional Cash Transfer (CCT) programme targeting pregnant women in rural and underserved areas. The potential benefits of this approach to overcoming barriers to access and saving lives should be considered in light of this report. This paper intends to provide a comprehensive overview of the steps taken and the lessons learnt in implementing the pilot phase in this complex setting, and draw on available data to monitor the demand for basic health services.

### Background and rationale for SURE-P MCH conditional cash transfer

The poor MNCH outcomes observed in Nigeria are partly attributable to the low coverage and uptake of basic health interventions that would be effective in preventing maternal and neonatal deaths. Thirty-nine (39%) percent of pregnant women received no antenatal care (ANC), and only 38.1% of mothers delivered with a skilled provider [1]. These averages disguise wide variations across the country by geographical and socioeconomic characteristics: the percentage of deliveries with a skilled attendant varies from 12.3% in the North West geopolitical zone to 82.5% in the South West. Overall, 22.7% of women in rural areas deliver with a skilled attendant, as against 67.0% in urban areas [1]. These patterns are replicated in other key indices of reproductive and child health, which also show highly inequitable healthcare coverage and outcomes by household wealth [4]. Strategies for improving these indices by the Federal Government of Nigeria have focussed since 2009 on programmes that can achieve results, in terms of access to healthcare and improved health outcomes. This approach has been marked by the integration of new interventions with comprehensive impact evaluations, and most recently by the high-profile target of Saving One Million Lives (SOML) by 2015, integrating new and existing primary health care (PHC) activities under the SOML initiative [5].

Previous key programmes to address the inequitable coverage of basic health services for MNCH include the Midwives Service Scheme (MSS), launched in 2009. This engages unemployed, newly graduated and retired midwives to work in selected PHC facilities in rural communities and has been extensively described elsewhere [6]. However, this recruitment, and other supply-side interventions, does not directly tackle demand-side barriers pregnant women face which stop them accessing care. The reasons for the low uptake of critical services in many developing countries, including Nigeria, are complex and multifactorial, and can include low education in the necessity of antenatal care, lack of confidence in existing health providers, women’s lack of decision-making authority, and physical or financial inability to access health services [7,8]. Pregnant women are especially likely to suffer as a result of user charges for health services, due to the expense of obstetric care and the lower financial resources generally available to women [9]. In Nigeria, whilst many states operate a policy of free MNCH services, in practice formal and informal out of pocket payments are common and are combined with the costs of transportation [10,11].

To address these demand-side barriers, the Federal Government of Nigeria introduced a Conditional Cash Transfer for maternal and child health under the SURE-P MCH programme. CCTs are social programmes, conditioning regular payments to poor households on use of certain social services. CCT programmes have been an established instrument of social protection for over twenty years, particularly in Latin America, and are also increasingly used in Africa and other regions [12,13]. The inclusion of a CCT component in SURE-P reflects an intention to improve national social safety nets in Nigeria, in this case by using direct financial support to women in rural areas who are otherwise vulnerable to financial hardship when accessing care. The CCT programme is also part of a broader demand stimulation strategy by SURE-P MCH, which includes recruitment of Village Health Workers to work directly in targeted communities. The CCT programme is operating in a subset of PHCs supported by the SURE-P MCH Project, all of which receive supply-side upgrades in the form of infrastructure upgrades and equipment, commodities and human resources.

## Methods

### CCT pilot programme design

The CCT Programme provides financial incentives to women enrolling in the programme for attending key health services, to promote retention throughout the continuum of care with its associated health benefits [14] (see Table 1). In total, women may receive up to N 5,000 (approximately US$30), pro rated according to which co-responsibilities she completes. This value was arrived at by estimating the average cost incurred by pregnant women on out-of-pocket expenses and transport costs to health facilities, according to community surveys and focus group discussions conducted in 2012 during pre-pilot planning. This is of similar scale to other CCT programmes targeting maternal health [15].

**Table 1:** **List of CCT co-responsibilities**

**No.**	**Requirement**	**Value**	**Time collected**
1	Registration and attending first antenatal care consultation (ANC 1) – completed together	N 1000	After registration and verification of details at central database
2	At least three further ANC consultations (ANC 2, 3, 4)	N 1000 (pro rated)	After delivery and verified completion of programme
3	Delivery with skilled assistance (SBA)	N 2000	
4	First immunization for neonate, and/or post-natal visit with family planning advice for mother	N 1000	

Descriptions of the MNCH services a CCT beneficiary must attend to receive a cash stipend: the total amount available to each beneficiary is N 5,000.

The CCT operates geographic targeting, by making enrollment available to any woman attending PHCs participating in SURE-P (all selected by their location in rural and otherwise underserved communities), rather than directly targeting low-income groups through means assessment. This avoids the high administrative costs observed in cash transfer programmes that target by socio-economic characteristics, as this information is not readily available at the household level in Nigeria. Furthermore, the overall goal of the programme is improvement of maternal and child health, rather than poverty reduction, and therefore there is no need to restrict the CCT to women below a defined poverty line. CCT beneficiaries referred from the PHC to hospital at any stage in their pregnancy or delivery also receive free care for a defined package of benefits, reimbursed to the hospital by SURE-P.

Beneficiaries only receive money after their attendance of each service has been logged and verified, to increase public trust in the programme [13]. Data on CCT beneficiaries is initially collected at facilities by SURE-P’s trained CCT field staff, using the facility’s patient record files to check the co-responsibilities fulfilled by each woman enrolled, and sent to the central Project Implementation Unit (PIU). This data is used to calculate the amount to be paid to each woman. The expected amount is checked at the point of cash disbursement against the attendance dates recorded by PHC staff on her CCT beneficiary handcard, in order to verify compliance with conditions.

The Key Performance Indicators for the CCT programme and SURE-P MCH as a whole include facility attendance, clinical outcomes, and the programme’s operational efficiency. The following indicators are calculated on a weekly basis from data sent by field staff, to generate lists of the amounts due to each woman, and to monitor in real time the success of the programme at stimulating demand throughout the continuum of care:

1. Beneficiaries registering and attending their first antenatal care consultation (ANC 1)
2. Beneficiaries completing the minimum required antenatal care course to ANC 4
3. Beneficiaries delivering with Skilled Birth Attendance (SBA)
4. Beneficiaries returning to the facility for post-natal checks, family planning advice, and neonatal immunization.

These figures are monitored both in terms of total service use, and the percentage of beneficiaries who are retained between each stage of the continuum of care.

Before setting up the Pilot Programme, a six-week pre-pilot was held in two wards of the Federal Capital Territory (FCT) to gauge the interest of pregnant women and their communities in a future programme, and to test the effectiveness of data reporting tools and other operational mechanics. Programme operations, surveys and focus groups confirmed the appeal of the incentives both to women with and without a history of previously using health services.

### Implementation

**Figure 1:**
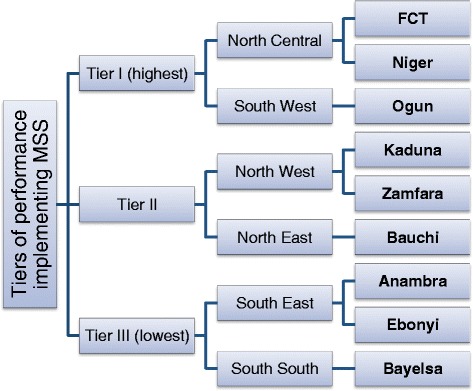
**States selected for CCT Pilot Programme.** List of the states selected for the Conditional Cash Transfer (CCT) Pilot Programme, shown on the right of the diagram. The figure shows how these states were selected: first by determining that three states should be represented from each of three tiers of performance in a previous evaluation of the Midwives Service Scheme (MSS) in 2012. For each tier, three states were selected from two of Nigeria’s six geopolitical zones. Abbreviations: FCT, Federal Capital Territory.

The CCT programme is initially being piloted in nine states. These were selected to provide representation from each of the six geo-political zones, and to include three states from each tier of performance in implementing the earlier MSS programme (assessed based on their improvement in key performance indicators), as shown in Figure 1. This cross-section was selected in order to allow for a comparative analysis of states’ experiences implementing the pilot. Each state operates a Steering Committee to oversee the programme, composed of government and civil society representatives from the state and the local government areas where the CCT programme is implemented. A cluster of four PHCs and one general hospital in each state was selected which had sufficient existing infrastructure and human resources for health to be able to handle the basic requirements of the pilot.

The CCT Pilot Programme began in 5 PHC facilities (one SURE-P cluster of four facilities and an additional non-SURE-P facility carried over from the pre-pilot) in the Federal Capital Territory (FCT) in April 2013. In July and August 2013, 28 facilities in 7 other states began implementing the programme, while the final cluster in Ogun State began implementation in September 2013. In total 9 clusters (36 SURE-P facilities^b^) are therefore implementing the CCT in its pilot stage. There are a further 22 clusters (88 SURE-P facilities) in the nine states in question, which serve as a comparison group for the analysis below.

### Data analysis

Routine monitoring data is available up to 31^st^ March 2014 from two sources in SURE-P MCH (Table 2). A consultation with the National Primary Health Care Development Agency’s Ethics Review Committee, prior to beginning the analysis, determined that ethical approval for analysis using this monitoring data was not required. However, permission was sought and approval obtained from the Committee to publish the analysis.

**Table 2:** **Data used for analysis**

**Source**	**Unit of data**	**Coverage**	**Start date**	**End date**	**Types of analysis made**	**Sections below**
**SURE-P MCH CCT Beneficiary database**	Individual	CCT facilities	April 2013	March 2014	Monitoring enrollment in CCT programme;	**Results**: Enrollment of beneficiaries;
					Comparison between clusters implementing CCT programme.	**Results**: Beneficiary retention through continuum of care
**SURE-P MCH M&E Data**	Facility	All SURE-P MCH	January 2012	March 2014	Comparison between SURE-P clusters implementing and not implementing the CCT programme.	**Results**: Impact on demand

The CCT beneficiary database, as collected by field staff, is used for monitoring beneficiary enrollment and compliance with the programme. Data from the CCT beneficiary database was summarised and graphed in Excel to calculate the total enrollment in each month of the programme, and the percentage of beneficiaries who had been retained through key points in the continuum of care.

Separate to this process, SURE-P MCH Monitoring and Evaluation (M&E) data on every facility under SURE-P is collected in quarterly cluster monitoring visits, and is subsequently subject to independent data quality assessment^c^. Monthly attendance figures were calculated using the primary source of facility logbooks, and collected by trained short-term enumerators who also delivered spot training on record keeping as required. This approach ensures that the main finding of interest – service uptake directly before and after the CCT programme began – is based on internally comparable data both between facilities and over time. These monthly attendance figures are entered in Excel by programme staff, and for this analysis were summed for each group of clusters to produce total attendance figures across the clusters in each month. Finally, the approximate service uptake was calculated as the total attendance from PHC registers, standardized per 100,000 catchment population (total catchment population in the 9 CCT clusters is 637,227; in 22 comparison clusters, 1,385,574).

The statistical significance of the time trends in attendance was tested using a segmented regression analysis. This approach allows an estimate of the extent to which changes in outcomes are due to the impact of a policy intervention, as opposed to unrelated secular trends [16]. A regression model was used in Stata 12.0 for each outcome variable with the form:

$$ {\mathrm{Y}}_{\mathrm{t}}={\upbeta}_0+{\upbeta}_1\mathrm{time} + {\upbeta}_{2\mathrm{a}}\mathrm{interventionSupply} + {\upbeta}_{2\mathrm{b}}\mathrm{interventionCCT} + {\upbeta}_{3\mathrm{a}}\mathrm{postslopeSupply} + {\upbeta}_{3\mathrm{b}}\mathrm{postslopeCCT} $$

In this model, Y_t_ is the total attendance for a given service (outcome) across a group of facilities at time t, per 100,000 catchment population; outcomes were selected which correspond to each of the four CCT co-responsibilities throughout the continuum of care (see Table 1). β_1_ estimates the trend in the outcome attributable to time, independent of any interventions. β_2a_ and β_2b_ represent immediate effects of the supply and CCT interventions respectively on the level of the outcome; the independent dummy variables interventionSupply and interventionCCT were coded zero or 1 for each month based on the dates each intervention was launched, with interventionCCT remaining zero throughout the dataset for the comparison clusters. β_3a_ and β_3b_ estimate the change in trend for the outcome following the launch of each intervention: supply-side upgrades from October 2012 and the CCT programme between April-September 2013 (as in Table 3 below). The respective postslope variables were coded zero in the months prior to each intervention launching, and sequentially from 1 thereafter. The time series for each cluster were aligned around the postslopeCCT variable, which varied according to launch date, and summed accordingly within each group of facilities (CCT clusters and comparison clusters).

**Table 3:** **Cumulative totals of selected indicators in Pilot Programme, as at 31.03.2014**

	**Apr 2013**	**May 2013**	**Jun 2013**	**Jul 2013**	**Aug 2013**	**Sep 2013**	**Oct 2013**	**Nov 2013**	**Dec 2013**	**Jan 2014**	**Feb 2014**	**Mar 2014**
**Number of participating PHC facilities**	4	5	5	30	33	37	37	37	37	37	37	37
**Number of women enrolled in CCT**	323	1,431	2,154	3,894	6,681	9,055	11,153	13,501	15,272	17,208	18,612	20,133
**States participating**	FCT			Anambra, Bauchi, Bayelsa, Ebonyi, FCT, Kaduna, Niger, Zamfara		Anambra, Bauchi, Bayelsa, Ebonyi, FCT, Kaduna, Niger, Ogun, Zamfara						

Data source: facility logbooks in PHCs implementing the CCT programme.

Durbin-Watson tests were performed after each regression to check for the need to control for first-order auto-correlation and use an alternative regression method. Based on the result of this test, a Prais-Winston regression was fitted to the trends in one outcome, namely the number of women attending four or more ANC consultations. Segmented regression using an ordinary least square approach was fitted for all other outcomes, as the Durbin-Watson tests did not indicate auto-correlation [16].

## Results

### Enrollment of beneficiaries

**Figure 2:**
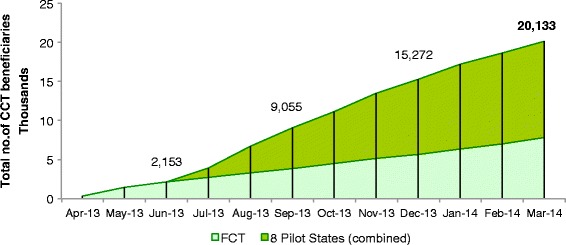
**Cumulative total enrollment of CCT beneficiaries, as at 31.03.2014.** Shows the number of women enrolled in the Conditional Cash Transfer (CCT) Pilot Programme since operations began in April 2013. The total beneficiary count at the end of each quarter and as at 31^st^ March 2014 are shown. The trend for the Federal Capital Territory (FCT) is shown as a subset of the overall cumulative total, as during the second quarter of 2013 this was the only state implementing the pilot.

Figure 2 and Table 3 show that by 31^st^ March 2014, a total of 20,133 women had enrolled as CCT beneficiaries. The rates of enrollment have been steady over time.

### Beneficiary retention through continuum of care (comparison among CCT clusters)

**Figure 3:**
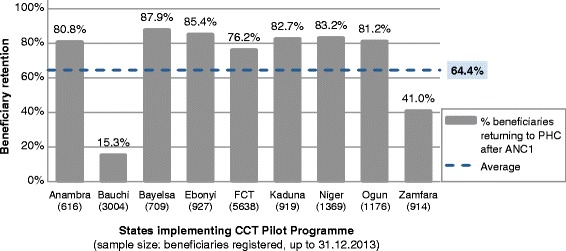
**Percentage of CCT beneficiaries observed returning after enrolment, by state, as at 31.03.14.** Shows the percentage of Conditional Cash Transfer (CCT) beneficiaries who were recorded by project staff as returning to the primary healthcare (PHC) facility they enrolled at. Returning is defined as fulfilling any of co-responsibilities 2–4 in Table 1. The denominator (number of beneficiaries in sample) is shown below each state. Abbreviations: ANC, Antenatal care.

Figure 3 shows that overall, 64.4% of CCT beneficiaries registering in 2013 (from the programme start date, per Table 3, to 31^st^ December 2013) were observed returning to the facility at least once before 31^st^ March 2014. The percentages vary strikingly by state; between 87.9% (in Bayelsa State) and 15.3% (in Bauchi State) of beneficiaries were recorded as returning after registration.

**Figure 4:**
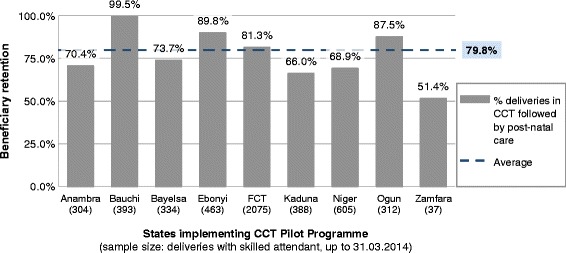
**Percentage of deliveries which were followed by post natal care, by state, as at 31.03.14.** Shows the percentage of Conditional Cash Transfer (CCT) beneficiaries who were recorded by project staff as returning to the primary healthcare (PHC) facility after delivery. Returning is defined as fulfilling co-responsibility 4 in Table 1: either by attending for zero-dose neonatal immunization, or for a post-natal visit for the mother, or both. The denominator (number of deliveries in sample) is shown below each state.

The sample of beneficiaries who have been in the programme for the full duration of a pregnancy is presently too small to analyse retention over the full continuum of care in detail. However, one aspect of retention that can be accurately observed from the current datasets is between delivery and post natal follow up. Figure 4 shows the percentage of deliveries in the Pilot Programme that were followed up by post natal care and/or immunizations, which should occur within one week of delivery. In total 79.8% of beneficiaries who delivered returned to the facility for follow-up; again, the performance in each state varies, from 51.4% of mothers returning in Zamfara State to 99.5% in Bauchi State.

### Impact on demand (comparison between CCT and non-CCT facilities)

**Figure 5:**
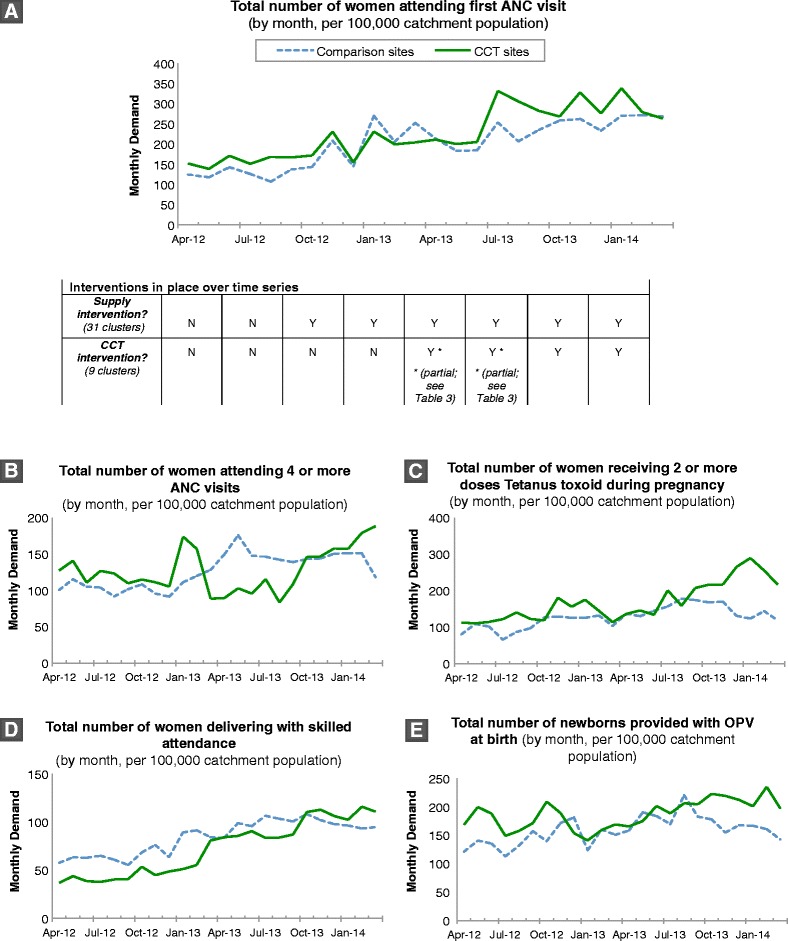
**Time series plot of monthly service uptake for key services, in states piloting CCT programme.** Trends in service use at 31 clusters in nine states piloting the SURE-P Conditional Cash Transfer (CCT), shown as monthly totals standardised per 100,000 catchment population from 2012–2014. The trends are shown separately for the nine clusters implementing the CCT and 22 comparison clusters. **A)** Total attendance for first antenatal care visit; **B)** Total number of pregnant women attending four or more ANC visits; **C)** Total number of women receiving two or more doses Tetanus toxoid during pregnancy. **D)** Total number of women delivering with skilled attendance; **E)** Total number of newborns provided with zero-dose OPV. Abbreviations: ANC, Antenatal care; OPV, Oral polio vaccine.

Figure 5 shows demand for the services tracked as Key Performance Indicators for the CCT, using data collected by SURE-P MCH M&E to compare effectively with non-intervention areas. These figures show time series trends in service uptake in the states piloting the CCT programme, comparing between the clusters implementing CCT and the comparison clusters implementing only a standard package.

Table 4 summarises these time trends as modelled in the segmented regression analysis for each outcome, which is represented as attendance for each of the services tracked, per 100,000 catchment population. This table shows coefficients, confidence intervals and statistical significance of the trends in both sets of clusters, and isolates the estimated impact of both the supply and CCT interventions sequentially. The statistically significant results are described below.

**Table 4:** **Parameter estimates, t-statistics and confidence levels for services along continuum of care**

**Models and variables**		**Coefficient**	**t-stat**	**P-value**	**95% confidence interval**
**Women attending first ANC visit (Segmented regression)**					
Comparison clusters	*β*_*0*_ *: Intercept*	124.3333***	4.42	0.000	(65.50, 183.17)
	*β*_*1*_ *: Secular (time) trend*	0.4145	0.06	0.955	(−14.69, 15.52)
	*β*_*2a*_ *:* *Supply effect on level*	48.7914	1.83	0.083	(−7.07, 104.65)
	β_*3a*_ *:* *Supply effect on trend*	4.8908	0.66	0.515	(−10.54, 20.32)
CCT clusters	*β*_*0*_ *: Intercept*	129.5687***	4.74	0.000	(71.88, 187.254)
	*β*_*1*_ *: Secular (time) trend*	7.5109	1.07	0.300	(−7.30, 22.32)
	*β*_*2a*_ *:* *Supply effect on level*	−12.0445	−0.41	0.685	(−73.71, 49.62)
	*β*_*3a*_ *:* *Supply effect on trend*	0.0031	0.00	1.000	(−16.30, 16.31)
	***β***_***2b***_ ***: CCT effect on level***	**59.2698**	**1.96**	**0.067**	**(−4.52, 123.06)**
	***β***_***3b***_ ***: CCT effect on trend***	**−8.3150**	**−1.29**	**0.213**	**(−21.87, 5.24)**
**Women attending at least 4 ANC visits (Prais-Winston regression)**					
Comparison clusters	*β*_*0*_ *: Intercept*	103.5899***	6.06	0.000	(67.83, 139.35)
	*β*_*1*_ *: Secular (time) trend*	−0.2366	−0.06	0.951	(−8.26, 7.79)
	*β*_*2a*_ *:* *Supply effect on level*	4.5210	0.36	0.726	(−22.08, 31.12)
	*β*_*3a*_ *:* *Supply effect on trend*	3.2709	0.76	0.456	(−5.72, 12.26)
CCT clusters	*β*_*0*_ *: Intercept*	99.9540***	6.30	0.000	(66.51, 133.40)
	*β*_*1*_ *: Secular (time) trend*	3.4686	0.85	0.408	(−5.17, 12.10)
	*β*_*2a*_ *:* *Supply effect on level*	13.0980	0.77	0.454	(−22.93, 49.13)
	*β*_*3a*_ *:* *Supply effect on trend*	−5.1937	−1.17	0.259	(−14.57, 4.18)
	***β***_***2b***_ ***: CCT effect on level***	−45.53212*	**−2.58**	**0.019**	**(−82.71, −8.36)**
	***β***_***3b***_ ***: CCT effect on trend***	15.1152**	**4.13**	**0.001**	**(7.38, 22.85)**
**Women receiving at least 2 doses Tetanus toxoid (Segmented regression)**					
Comparison clusters	*β*_*0*_ *: Intercept*	92.0673***	5.23	0.000	(55.26, 128.88)
	*β*_*1*_ *: Secular (time) trend*	−0.6289	−0.14	0.891	(−10.08, 8.82)
	*β*_*2a*_ *:* *Supply effect on level*	34.2304	2.05	0.054	(−0.72, 69.18)
	*β*_*3a*_ *:* *Supply effect on trend*	2.6467	0.57	0.573	(−7.00, 12.30)
CCT clusters	*β*_*0*_ *: Intercept*	119.5147***	4.77	0.000	(66.62, 172.41)
	*β*_*1*_ *: Secular (time) trend*	−1.7751	−0.28	0.786	(−15.36, 11.81)
	*β*_*2a*_ *:* *Supply effect on level*	45.8895	1.71	0.105	(−10.66, 102.44)
	*β*_*3a*_ *:* *Supply effect on trend*	1.7798	0.25	0.805	(−13.17, 16.73)
	***β***_***2b***_ ***: CCT effect on level***	**−19.01**	**−0.69**	**0.502**	**(−77.51, 39.48)**
	***β***_***3b***_ ***: CCT effect on trend***	21.65**	**3.68**	**0.002**	**(9.23, 34.08)**
**Women delivering with skilled attendance (Segmented regression)**					
Comparison clusters	*β*_*0*_ *: Intercept*	62.5497***	8.32	0.000	(46.82, 78.28)
	*β*_*1*_ *: Secular (time) trend*	−0.4847	−0.25	0.804	(−4.52, 3.55)
	*β*_*2a*_ *:* *Supply effect on level*	15.0429*	2.11	0.049	(0.11, 30.00)
	*β*_*3a*_ *:* *Supply effect on trend*	2.37	1.20	0.244	(−1.76, 6.49)
CCT clusters	*β*_*0*_ *: Intercept*	37.8933***	6.72	0.000	(26.00, 49.80)
	*β*_*1*_ *: Secular (time) trend*	0.0314	0.02	0.983	(−3.02, 3.09)
	*β*_*2a*_ *:* *Supply effect on level*	−0.0332	−0.01	0.996	(−12.75, 12.68)
	*β*_*3a*_ *:* *Supply effect on trend*	4.8661**	3.05	0.007	(1.50, 8.23)
	***β***_***2b***_ ***: CCT effect on level***	**−8.2210**	**−1.32**	**0.205**	**(−21.38, 4.93)**
	***β***_***3b***_ ***: CCT effect on trend***	**0.6618**	**0.50**	**0.624**	**(−2.13, 3.46)**
**Newborns provided with OPV at birth (Segmented regression)**					
Comparison clusters	*β*_*0*_ *: Intercept*	120.0659***	6.35	0.000	(80.47, 159.67)
	*β*_*1*_ *: Secular (time) trend*	3.7302	0.77	0.452	(−6.44, 13.90)
	*β*_*2a*_ *:* *Supply effect on level*	15.1527	0.84	0.409	(−22.44, 52.75)
	*β*_*3a*_ *:* *Supply effect on trend*	−2.5497	−0.51	0.613	(−12.93, 7.83)
CCT clusters	*β*_*0*_ *: Intercept*	155.8673***	14.71	0.000	(133.51, 178.22)
	*β*_*1*_ *: Secular (time) trend*	3.8689	1.42	0.173	(−1.87, 9.61)
	*β*_*2a*_ *:* *Supply effect on level*	−24.2458*	−2.14	0.047	(−48.14, −0.35)
	*β*_*3a*_ *:* *Supply effect on trend*	−0.6203	−0.21	0.838	(−6.94, 5.70)
	***β***_***2b***_ ***: CCT effect on level***	**1.1507**	**0.10**	**0.923**	**(−23.57, 25.87)**
	***β***_***3b***_ ***: CCT effect on trend***	**3.0739**	**1.23**	**0.234**	**(−2.18, 8.33)**

Note: ***P < 0.001, **P < 0.01, *P < 0.05.

Effects of the CCT intervention in the regression model are shown in **bold text**.

Data source: facility logbooks in the 124 PHCs where data was collected.

### First ANC visit

The time series in Figure 5(A) show gradual increases in attendance at all facilities, with an apparent additional rise in demand after the CCT was added to the SURE-P package of interventions. The regression analysis, however, did not find effects of either the supply or CCT interventions to be significant at the 5% level in either group of facilities.

### Multiple ANC visits

The time trends for women recorded attending four or more ANC visits (Figure 5(B)) show irregular attendance over time at all facilities, with an unexplained fall in attendance in Q2-Q3 2013 among the CCT facilities and a subsequent recovery. Prais-Winsten regression found no significant effects of the supply intervention, in either group of facilities. However, effects were observed from the CCT intervention: total attendance actually fell immediately after the programme began by 45.53 consultations per 100,000 catchment population (95% CI: −82.71 to −8.37), significant at the 5% level. The monthly average, however, increased following the introduction of CCT by 15.11 visits per 100,000 catchment population per month (95% CI: 7.38 to 22.85), significant at the 0.1% level.

The retention of women through the ANC continuum of care is also approximated by the outcome for women receiving at least two doses Tetanus toxoid injection, also shown in Figure 5(C). The time trend here shows apparently stationary attendance over time in the comparison facilities and an indistinct but gradual increase over time in CCT facilities. Segmented regression found no significant effects of the supply intervention, in either group of facilities. A slope effect alone was observed from the CCT intervention: the monthly average increased following the introduction of CCT by 21.66 cases per 100,000 catchment population per month (95% CI: 9.23 to 34.08), significant at the 1% level.

### Delivery with skilled attendance

Time trends (Figure 5(D)) shows an irregular but apparently non-stationary increase in demand over time in both groups, which is more pronounced in the CCT facilities. In spite of this, segmented regression does not detect a significant level or slope effect following the CCT intervention. The facilities implementing the CCT progamme, however, demonstrate a statistically significant slope effect following the supply intervention, with an increase of 4.87 deliveries per 100,000 catchment population per month (95% CI: 1.50 to 8.23). In the comparison facilities, an statistically significant level increase was observed after the supply intervention began, of 15.04 deliveries per 100,000 catchment population (95% CI: 0.11 to 30.00). Despite the visual inspection of trends suggesting non-stationarity, the secular increase over time (β_1_) is also not statistically significant in either group of facilities.

### Neonatal immunization upon post-natal attendance

This is shown using records of service uptake for the first oral polio vaccine (OPV) dose to neonates. The time trends for this outcome (Figure 5(E)) again show fluctuating demand with slight overall increases over time. Segmented regression did not show significant effects in the comparison group; in the group implementing the CCT programme, there were also no significant effects following the launch of the CCT. However, in the CCT group a statistically significant one-off drop in attendance was observed following the introduction of the supply intervention, of 24.24 vaccines provided per 100,000 catchment population (95% CI: −48.14 to −0.35).

## Discussion

### Impact observed

The CCT Pilot Programme, as observed in its early stages, generates positive results in the regression analysis for two outcomes. Significant positive slope effects were observed after the programme began on the number of women attending for four or more ANC visits, despite an initial negative level effect, and women receiving adequate Tetanus toxoid doses. Although the official dose schedule does not require that a woman attends four ANC appointments to receive two doses, it does require repeated visits, and therefore if a trend is observed for the fourth ANC visit, it would also be expected for this weaker condition [17].

The facilities implementing the CCT programme also showed a significant rise after the supply intervention began on the monthly rates of delivery with skilled attendance, although there was also a level decrease in neonatal immunization. The effects in the regression of the supply intervention are not consistent between the two groups of facilities, either in terms of coefficient value or statistical significance.

Some trends in demand fluctuated more than expected, in particular a dip in the recorded number of women attending four or more ANC visits between March-September 2013, the reason for which is not clear but does not appear to be seasonal. The statistically significant negative level effect on this outcome after the CCT was introduced is probably due to this drop. The initial estimated demand (per 100,000 catchment population) was slightly higher for all indicators in the CCT facilities than in the comparison group, which may be due to the purposive selection of facilities for the CCT as those which were able to handle the operational requirements of a programme pilot.

This analysis was guided by a desire to monitor the service uptake throughout the continuum of care, as the CCT programme was designed to promote this behaviour change in women enrolling. The analysis both within the CCT beneficiary database and at the facililty level shows that continual effort is required to stimulate attendance and improve data collection. Loss of CCT beneficiaries to follow-up remains a challenge, with over 35% of beneficiaries not returning after initially enrolling. This suggests failings either by PHCs to track beneficiaries and encourage them to return to the facility, or by record-keeping staff logging return visits. However, this figure cannot be directly compared to any baseline, so we cannot yet say if the challenges faced here are similar to those in other programmes. The differences in performance between clusters in terms of retaining beneficiaries throughout the continuum of care needs to be explored further. Performance far outside the norm is being investigated to find out if apparently poor results are due to partial data capture. This may be the reason for the high observed attrition in Bauchi state, and the lower-than-expected enrollment of beneficiaries in Zamfara.

### Implementation processes

The experience to date implementing the CCT Pilot Programme is in line with previous findings that CCT programmes can work to increase demand for health services, including MNCH services, although they are not a panacea [18,19]. The early results are also encouraging in terms of process evaluation, as they demonstrate large-scale programme operations which can soon be expected to reach even more beneficiaries than the last major CCT in Nigeria, In Care of the People (COPE) [20]. The major lessons learned from the early implementation phase are:

- The requirements for successfully operating a cash transfer programme includes contractual relationships with a number of other bodies, including information system developers and local banks or other financial institutions, to undertake key administrative functions. These relationships need to be defined at an early stage and monitored throughout the programme.
- The additional demand generated and some new reporting tools created additional workload for the participating facilities, which had varying levels of capacity to handle this work. Facility staff may need to be compensated for these additional demands.
- Prompt cash transfers are an essential part of the programme, to build trust and for the cash to serve as an intended counter to the costs of healthcare for mothers.
- Monitoring the programme uptake and performance in each implementing cluster is essential, in order to track and address outcomes which may be the result of poor data capture or of operational barriers.
- Start-up costs in each state can be expected to be high, covering formative research and advocacy, development of new management information systems to track beneficiaries on an individual level, engagement of additional field staff for data collection, and logistics and security for cash disbursement events. However, this would also be the case for other demand-side financing schemes, all of which require significant investment in administrative and management structures [21].

The general theme of these lessons, which has been echoed in previous case studies [22], is that even a conceptually simple demand-side financing scheme requires significant administrative structures and may be subject to bottlenecks at various levels. Issues that are particularly important for programme implementation in the Nigerian context, and are included in the design of this programme, include the integration of demand-side financing with supply-side improvements. For improved access to services to be translated into improved outcomes, the services must be sufficiently resourced and good quality to have the intended health effect [20].

### Limitations of data

In the regression analysis, the number of months since the programme began (the *postslope* variable) is relatively small: although this model can be applied with scarce longitudinal data, shorter time series unsurprisingly entail greater vulnerability to short-term fluctuations in outcomes [16]. In this case, the postslope variable for the CCT intervention only goes up to seven months (from the launch in Ogun State in September 2013 to the end of the dataset in March 2014). Given that many outcomes in this analysis (such as delivery) are not immediately responsive to policy change and only occur 3–6 months after women first attend the facility, it will be instructive to compare the trends at this early stage of the programme to the trends which emerge with a longer time-series.

Records of service uptake were standardized with reference to the total catchment area of each facility, due to a lack of available demographic and fertility data at the facility level. This could otherwise have been used to estimate number of expected births in each facility and thereby show total service coverage. The catchment area figures are collected at a single point in time and the denominator is therefore constant throughout the sample, with no available data on changes in the size or composition of the population. A seasonal variable was also not included in this analysis, as there was no previous evidence in these communities leading us to expect pregnancies or service use to vary by month.

The comparisons between CCT and non-CCT facilities implicitly assume that in the absence of the CCT intervention, all facilities sampled would have behaved in the same way and experienced the same trends in demand [23]. However, as the coefficients and significance of effects attributed to the common supply intervention differed between the two groups of facilities, it is feasible that this assumption would not hold. As the selection of facilities to implement the CCT programme was not randomised in this phase of the programme, there is a potential for systematic differences between the groups. Some potentially confounding factors are differences in facility characteristics such as the remoteness of facilities (supply factor) or female education levels in the community (demand factor) [23]. These factors are frequently static over the short to medium term, and are therefore less likely to influence the time trends observed within each set of clusters, which is the main subject of analysis in the regressions performed. At present, given the rural and previously under-documented nature of the facilities in this programme, there is a general lack of routine information available at the facility level about potentially confounding factors, other than previous utilisation. This is a problem common to social programmes in low and middle-income countries with acknowledged weaknesses in the health system and other social systems. The issue is being addressed in this programme by ongoing impact evaluation research, which is engaged in dedicated data collection at the level both of facilities under SURE-P MCH and the communities they are situated in, and is due for publication in 2015–2016 [5,24].

Although the data from 2012–2014 used for comparisons was collected using the same procedures across all PHCs, the quality of data could have improved differentially between the intervention and non-intervention groups as field staff are present more frequently in the CCT facilities.

## Conclusions

This overview of the implementation processes in a large national pilot has demonstrated some significant effects on outcomes of interest, although many outcomes were inconclusive. The purpose of this paper was to provide lessons from this programme which can be of use elsewhere, and the above discussion details specific implementation issues which are of interest in qualitative research. Although clusters implementing the Pilot Programme were not selected randomly, due to the operational requirements of starting a pilot, scale-up in 2014 includes random assignment of clusters to generate more robust comparisons.

The priority indicators reported above comprise a subset of the indicators tracked by the SURE-P MCH Project; evidence generated from ongoing programme implementation will be further examined in future monitoring and evaluation. An external Impact Evaluation will also use household and facility surveys to compare health service use, expenditure on healthcare, and health outcomes in clusters implementing the different components of SURE-P MCH [5,24].

This intervention, which has already reached over 20,000 women in its pilot phase, should be monitored along with other active CCTs for MNCH around the world. This will add to the growing body of work on the impact and best practice of demand-side interventions. The early lessons from implementing the programme, including experiences on cash delivery and infrastructural requirements, also have direct relevance for efforts in Nigeria to create and strengthen social safety nets.

## Endnotes

^a^Estimates apply national age-sex population distribution (from Demographic and Health Survey) to a projected 2013 population of 173.6 million.

^b^The comparative analysis includes 36 facilities in the intervention group, rather than 37 as described in ‘Implementation’ above: this is because one facility implementing the CCT is outside the SURE-P cluster system and therefore was not visited to collect the SURE-P MCH M&E data used.

^c^The Data Quality Assessments are conducted after each quarterly monitoring exercise, by a separate department in the National Primary Health Care Development Agency.

## Abbreviations

ANC: Antenatal care
CCT: Conditional cash transfer
FCT: Federal capital territory
M & E: Monitoring and evaluation
MNCH: Maternal, neonatal and child health
MSS: Midwives service scheme
OPV: Oral polio vaccine
PHC: Primary healthcare facility
PIU: Project implementation unit
SBA: Skilled birth attendance
SOML: Saving one million lives
SURE-P: Subsidy reinvestment and empowerment programme
